# Syndrome de McCune-Albright : à propos d’un cas et revue de la littérature

**DOI:** 10.11604/pamj.2023.46.33.35274

**Published:** 2023-09-22

**Authors:** Hédi Chabouni, Mohamed Ben Jemaa, Mohamed Ghorbel, Moez Trigui, Wassim Zribi, Mohamed Zribi, Kamel Ayadi, Mourad Aoui, Hassib Keskes

**Affiliations:** 1 Service de Chirurgie Orthopédique et Traumatologique, Centre Hospitalier Universitaire Habib Bourguiba Sfax, Sfax, Tunisie,; 2 Université de Sfax, Faculté de Médecine de Sfax, Sfax, Tunisie

**Keywords:** Dystrophie fibreuse, tache café au lait, puberté précoce, mutation, cas clinique, Fibrous dystrophy, café au lait task, precocious puberty, mutation, case report

## Abstract

Le syndrome de McCune-Albright est une maladie héréditaire caractérisée par l'association d'une dystrophie fibreuse de l'os, des taches cutanées café au lait et d'une puberté précoce révélant une hyperactivité endocrinienne. Sur le plan génétique, cette maladie est due à une mutation de la protéine Gs responsable d'une activation de l'adénylate cyclase avec production excessive de l'AMPc. La morphologie particulière des taches café-au-lait doit évoquer précocement le diagnostic. Son traitement dépend de l'endocrinopathie dont souffre le patient et de l'étendue de la dysplasie fibreuse. Les bisphosphonates ont prouvé leur efficacité sur les douleurs osseuses et la limitation de la dysplasie fibreuse. La chirurgie garde sa place dans les formes compliquées. Nous rapportons un cas rare d'un syndrome de McCune-Albright compliqué d'une fracture du fémur chez une fille de 12 ans et nous mettons au point les caractéristiques cliniques et paracliniques de cette entité pathologique.

## Abstract (en)

McCune-Albright syndrome is an inherited disease characterized by the association of fibrous dystrophy of bone, café-au-lait skin spots and precocious puberty revealing endocrine hyperactivity. Genetically, this disease is due to a mutation of the Gs protein responsible for activation of adenylate cyclase with excessive production of cAMP. The particular morphology of café-au-lait spots should suggest early diagnosis. Its treatment depends on the endocrinopathy from which the patient suffers and the extent of the fibrous dysplasia. Bisphosphonates have proven their effectiveness on bone pain and the limitation of fibrous dysplasia. Surgery retains its place in complicated forms. We report a rare case of McCune-Albright syndrome complicated by a femur fracture in a 12-year-old girl and we discuss the clinical and paraclinical characteristics of this pathological entity.

## Introduction

Le syndrome de McCune-Albright englobe des endocrinopathies dont la plus fréquente est la puberté précoce d'origine ovarienne, une dystrophie fibreuse des os et des taches café-au-lait de disposition blaschko-linéaire. L'étiopathogénie génétique a été décrite initialement par *Happle* en signalant son pouvoir létal à l'état homozygote. À travers un cas rare d'une dysplasie du fémur révélant une maladie de *McCune-Albright*, nous mettons au point cette pathologie rare et nous précisons ses particularités cliniques et paracliniques.

## Patient et observation

**Informations relatives aux patients:** une fille âgée de 12 ans, sans antécédents médicochirurgicaux et sans notion de consanguinité, consultait pour des douleurs isolées de la cuisse gauche sans notion de traumatisme et sans retentissement fonctionnel, ni déformation. Il n'y avait pas d'antécédents familiaux particuliers. A l'interrogatoire, la notion d'une puberté précoce a été découverte (ménarche à 10 ans).

**Figure 1:**
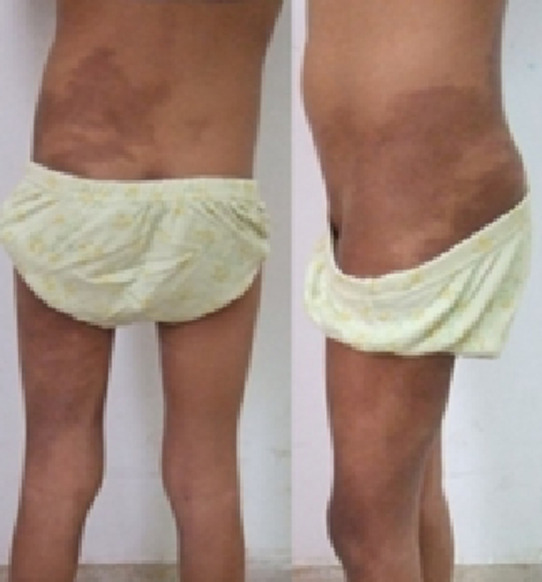
aspect clinique de taches cutanées café au lait diffuses au niveau du tronc et de la cuisse gauche

**Résultats cliniques:** à l'examen clinique, l'état général de l'enfant était conservé, on trouvait des taches cutanées café au lait diffuses au niveau de son tronc (Figure 1), par ailleurs, pas de neurofibromes cutanés.

**Figure 2:**
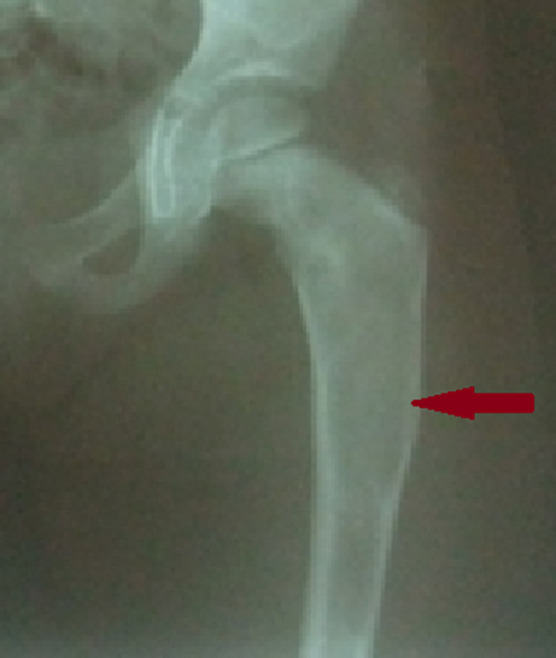
radiographie standard de la hanche de face révélant une lésion ostéolytique de l'extrémité supérieure du fémur gauche

**Démarche diagnostique:** la radiographie standard du fémur gauche révélait une lésion ostéolytique de l'extrémité supérieure du fémur gauche (Figure 2). Une dysplasie fibreuse osseuse de l'extrémité supérieure du fémur a été mise en évidence après examen anatomopathologique d'une biopsie chirurgicale. Le diagnostic positif était retenu devant la coexistence de la triade: puberté précoce, dysplasie fibreuse des os, taches café au lait.

**Figure 3:**
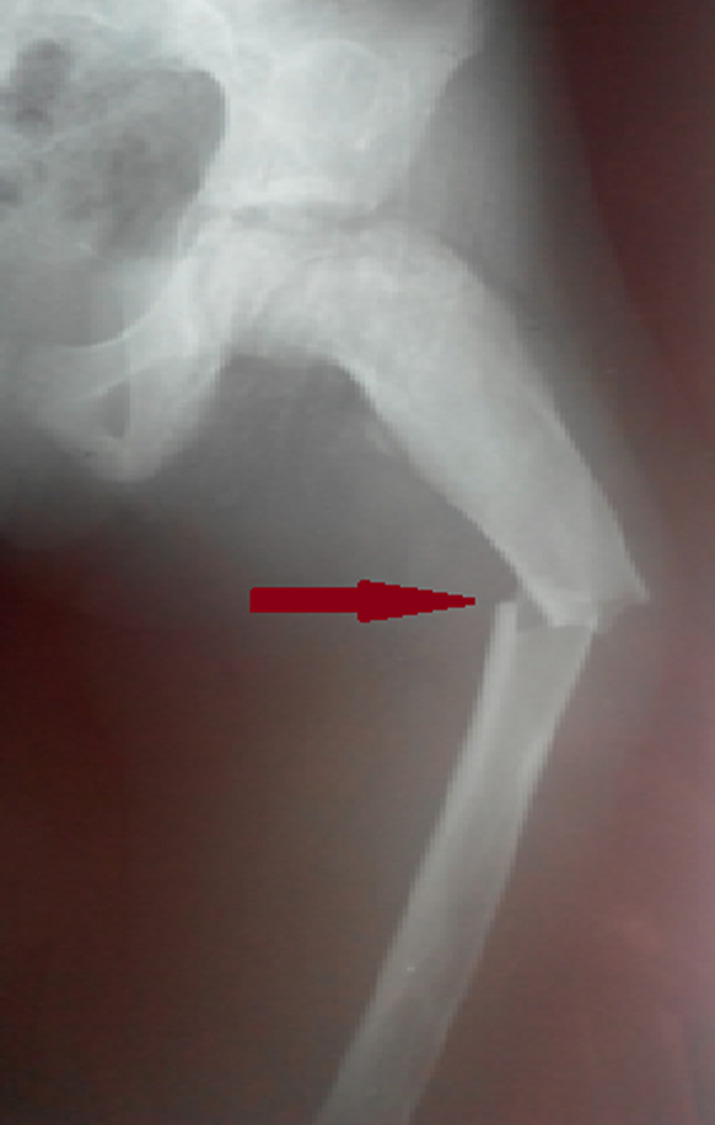
radiographie standard objectivant la fracture sous trochantérienne du fémur

**Figure 4:**
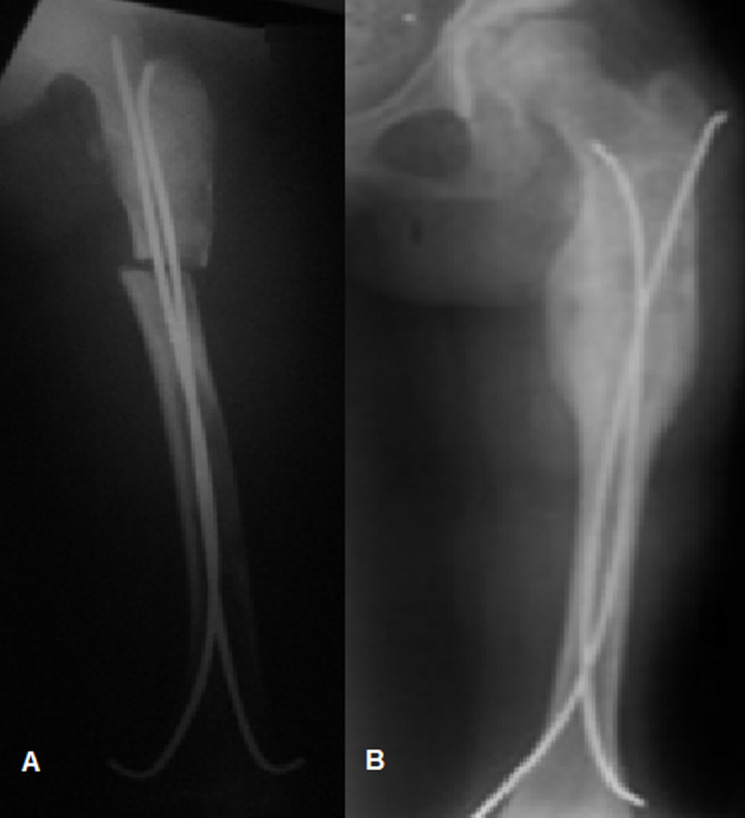
radiographie standard du fémur de face ; A) en post opératoire immédiat; B) à 3 mois post opératoire

**Intervention thérapeutique:** cette patiente a eu une surveillance régulière avec interdiction des efforts physiques importants. A l'âge de 9 ans, cette dysplasie fibreuse a été compliquée d'une fracture suite à un traumatisme bénin (Figure 3). Elle a eu un embrochage (Figure 4A).

**Suivi et résultats des interventions thérapeutiques:** la fracture était bien consolidée au bout de 3 mois (Figure 4B) et la reprise de la marche normale a été obtenue.

**Consentement éclairé:** les parents de la patiente ont donné leur consentement.

## Discussion

Le syndrome de McCune-Albright était décrit en premier en 1936 par McCune puis par Albright en 1937. Il représente une pathologie héréditaire rare initialement définie par l'association d'une triade: dysplasie fibreuse des os, taches café au lait et puberté précoce [1]. Ce syndrome est lié à une mutation somatique activatrice stimulant l'adénylate cyclase. Il en résulte un hyperfonctionnement des différents tissus atteints. Actuellement, cette définition englobe d'autres maladies endocriniennes pouvant être trouvées à côté de la puberté précoce telles que l'hyperthyroïdie, le syndrome de Cushing, … [2]. Des atteintes viscérales (cardiaques, hépatobiliaires et pancréatiques) ont été également rapportées [3,4].

La dysplasie fibreuse des os est la manifestation la plus constante de ce syndrome. L'atteinte peut être mono ou pluri-focale. Les principales localisations sont le massif facial, la base du crâne avec possibilité de compressions nerveuses optiques et auditives. L'atteinte du fémur proximal et du squelette axial est fréquemment rapportée. Elle peut être infra-clinique ou caractérisée par une boiterie, des douleurs, voire une fracture pathologique. Sur le plan anatomopathologique, cette lésion est caractérisée par le remplacement progressif de l'architecture normale de l'os par des cellules fusiformes avec une matrice de collagène désorganisé créant des zones kystiques entrainant une fragilisation de l'os. La radiographie standard montre un processus ostéolytique. L'évolution de la dysplasie fibreuse est incertaine. Elle subit habituellement une aggravation durant la croissance rapide avec risque de fractures et de déformations. Le risque de la transformation maligne est rare environ de 1%. La chirurgie est indiquée en cas de compression des nerfs crâniens, la survenue de fractures ou de déformations osseuses importantes ou persistance de douleurs rebelles au traitement médical signe d'alarme de survenue de complications. Le recours aux bisphosphonates semble avoir de bons résultats dans la prise en charge de la douleur. Néanmoins, l'efficacité reste encore discutée sur l'évolution de cette atteinte osseuse [5].

Les taches café-au-lait réalisent cliniquement des macules pigmentées sur une peau blanche. Sur le plan anatomopathologique, elle est due à une prolifération de macromélanosomes. Leur diagnostic est clinique. Elles sont parfois de petite taille à contour bien limité et d'une couleur brune. Elles sont des lésions congénitales qui évoluent avec la croissance. Les taches café-au-lait uniques ont une prévalence comprise entre 0,3 à 2,7 % chez les nouveaux nés. Leur fréquence évolue proportionnellement avec l'âge [6,7]. Les taches café au-lait multiples doivent faire évoquer une affection héréditaire en particulier la neurofibromatose de type 1. Dans le syndrome de McCune-Albright, les taches café-au-lait se distinguent par leur forme: cette forme est dite blaschko-linéaire. Elles sont segmentaires, souvent unilatérales. La puberté précoce atteint essentiellement les filles (30 à 50 %). Elle est due à un hyperfonctionnement ovarien indépendamment de la stimulation hormonale. Les anomalies thyroïdiennes sont la deuxième endocrinopathie la plus fréquente du syndrome de McCune-Albright (30 à 40 %). Le traitement dépend de l'endocrinopathie et de l'étendue de la dysplasie fibreuse [8,9]. Un diagnostic moléculaire semble être nécessaire pour l'approche diagnostique surtout devant une tache café-au-lait typique isolée. Cependant, un résultat négatif n'exclut en aucun cas le diagnostic d'où la nécessité d'un suivi régulier [10].

## Conclusion

Le syndrome de McCune-Albright est une pathologie héréditaire rare. Le diagnostic de ce syndrome est avant tout clinique. Le pronostic fonctionnel peut être mis en jeu à cause des douleurs, des déformations osseuses et du risque fracturaire. Les atteintes asymptomatiques nécessitent essentiellement un suivi régulier. La prise en charge de ce syndrome est multidisciplinaire associant des orthopédistes et des endocrinologues pour traitement spécifique de l'endocrinopathie. Les biphosphanates ont démontré leur efficacité dans le traitement des douleurs osseuses et limitation de l'étendue lésionnelle. La chirurgie garde ses indications dans les formes compliquées.
